# Clinical outcomes of pembrolizumab therapy in advanced‐NSCLC patients with poor performance status (≥3) and high PD‐L1 expression (TPS ≥50%): A case series

**DOI:** 10.1111/1759-7714.13713

**Published:** 2020-10-21

**Authors:** Ryoko Inaba‐Higashiyama, Tatsuya Yoshida, Hitomi Jo, Masayuki Shirasawa, Noriko Motoi, Yuichiro Ohe

**Affiliations:** ^1^ Department of Thoracic Oncology National Cancer Center Hospital Tokyo Japan; ^2^ Department of Pathology and Clinical Laboratory National Cancer Center Hospital Tokyo Japan

**Keywords:** Non‐small cell lung cancer (NSCLC), pembrolizumab, poor performance status, tumor proportion score, very high programmed death‐ligand 1 (PD‐L1) expression

## Abstract

Pembrolizumab is the standard first‐line treatment for advanced non‐small cell lung cancer (NSCLC) with programmed death‐ligand 1 (PD‐L1) expression tumor proportion score (TPS) ≥50%. The benefit of pembrolizumab in patients with advanced NSCLC and poor performance status (PS ≥3) is limited, even when the tumor is PD‐L1‐expression‐positive. We retrospectively reviewed a total of four NSCLC cases with high PD‐L1 expression (TPS ≥50%) and poor PS. The only patient with very high PD‐L1 expression (TPS 100%) responded to pembrolizumab, but none of the three patients with high PD‐L1 expression (50%–80%) responded to pembrolizumab. In conclusion, pembrolizumab can serve as a treatment option for patients with poor PS, if PD‐L1 expression TPS is 100%.

## Graphical abstract

Pembrolizumab may be a treatment option in patients with poor PS, if TPS of PD‐L1 on tumor cells are 100%.

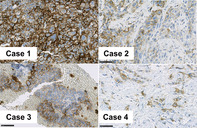

## Introduction

Pembrolizumab has been shown to have significantly greater efficacy than platinum doublet chemotherapy as a first‐line treatment for advanced non‐small cell lung cancer (NSCLC) patients with programmed death‐ligand 1 (PD‐L1) expression tumor proportion score (TPS) ≥50%.^1^ However, the efficacy of pembrolizumab in poor performance status (PS ≥3) advanced‐NSCLC patients with high PD‐L1 expression (TPS ≥50%) is still unclear.

**Figure 1:**
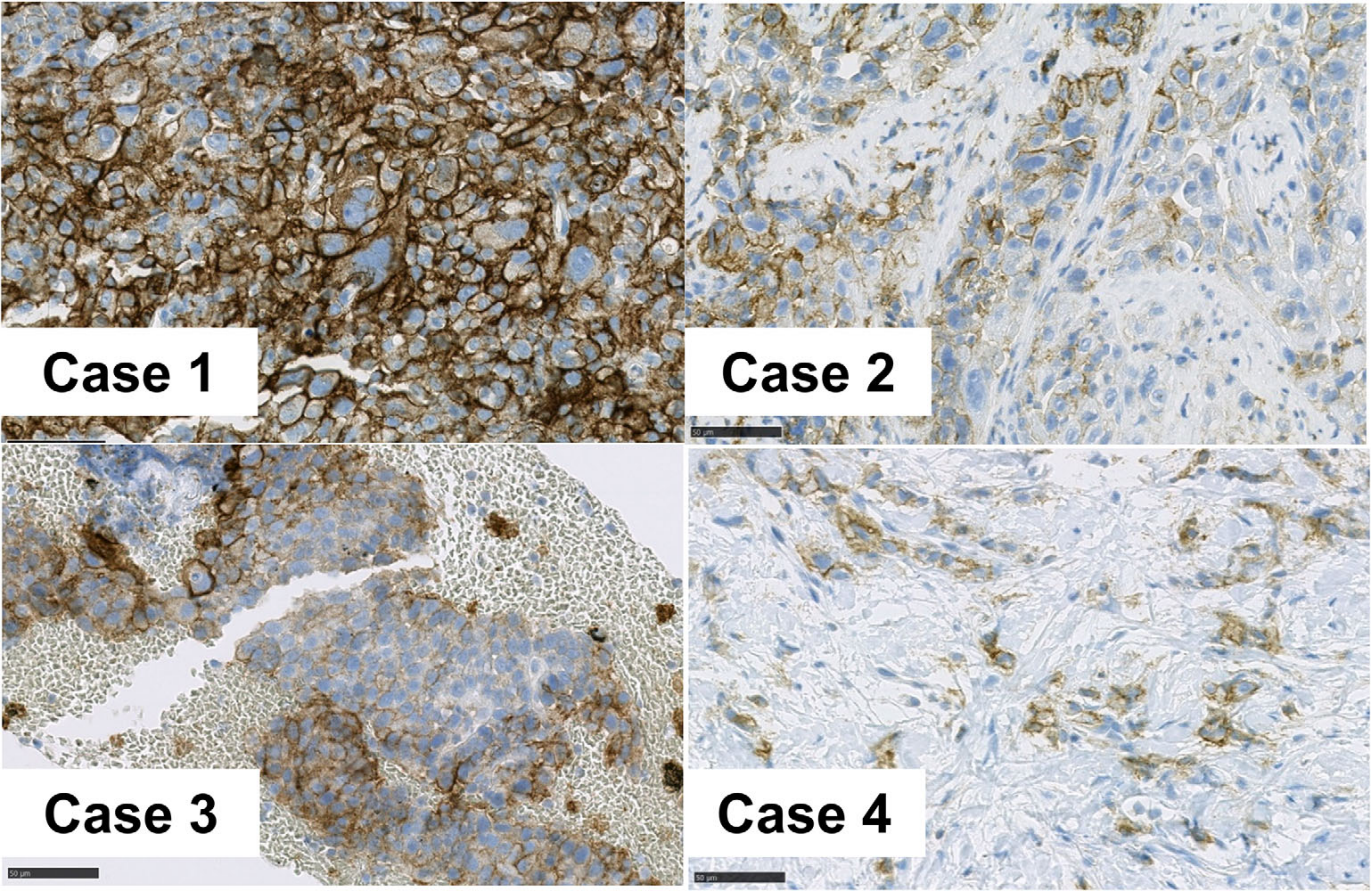
Immunohistochemical staining of tumor cells for PD‐LI (22C3) in the four cases. PD‐L1 expression was very high (TPS 100%) in Case 1 and more prominent than in the other three cases (Case 2–4).

Here, we retrospectively reviewed a total of 250 cases of advanced NSCLC treated with pembrolizumab as first‐line treatment at our hospital between May 2017 and December 2019 and identified a total of four patients with high PD‐L1 expression (TPS ≥50%) who had poor PS (≥3) and were driver‐mutation negative **(**Table 1 **)**. The assessment of PS was based on the patient's disease burden, and the PS of all four patients was 3. The TPS of PD‐L1 expression was 100% in one patient and 50%–100% in the other three patients **(**Fig 1 **)**. The patient (Case 1) with a very high level of PD‐L1 expression (TPS 100%) responded to pembrolizumab, but none of the three patients (Cases 2–4) with high PD‐L1 expression (50%–80%) responded to pembrolizumab. Here, we report the clinical course of the patient (Case 1) who responded to pembrolizumab.

**Table 1:** Clinical outcomes of four patients with advanced, strongly PD‐L1 ‐positive NSCLC treated with pembrolizumab

Case	Age (years)	Sex	PS	Histology	PD‐L1 TPS (%)	Best response	PFS (days)
1	60	Female	4	Adeno	100	PR	228+
2	76	Male	3	Adeno + pleo	50	PD	14
3	65	Male	3	Squamous	70	PD	21
4	78	Male	3	Adeno	80	PD	91

PS, performance status; TPS, tumor proportion score; PFS, progression‐free survival; Adeno, adenocarcinoma; pleo, pleomorphic carcinoma; Squamous, squamous carcinoma; PR, partial response; PD, progressive disease; SD, stable disease.

**Figure 2:**
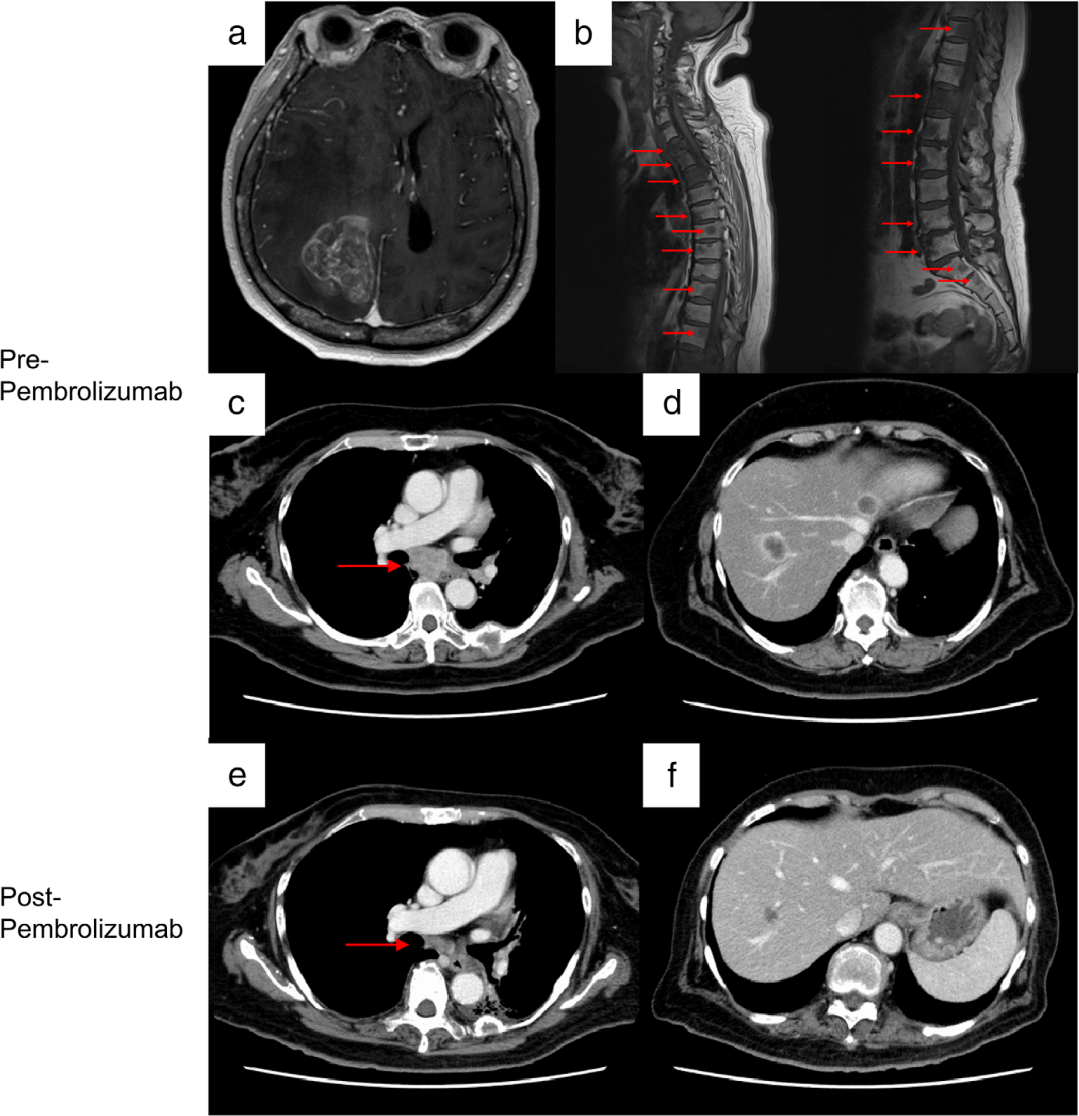
Brain and spinal cord MRI (contrast‐enhanced, T1‐weighted imaging) before pembrolizumab treatment showed (**a**) intratumoral bleeding from the brain metastasis in the right occipital lobe and (**b**) multiple bone metastases (red arrows). Chest and abdominal CT showed (**c** and **e**) mediastinal lymph node metastases (red arrows) and (**d** and **f**) liver metastases (red arrows) before and after pembrolizumab treatment.

## Case 1

A 60‐year‐old woman with a 40‐pack‐year smoking history was diagnosed with clinical stage IVB (T4N2M1c) NSCLC, not otherwise specified. The results of all driver oncogene tests, including epidermal growth factor receptor (*EGFR*) mutation, anaplastic lymphoma kinase (ALK) rearrangement, ROS proto oncogene 1 (ROS‐1) rearrangement, and v‐raf murine sarcoma viral oncogene homolog B (*BRAF*) mutation, were negative. PD‐L1 expression on tumor cells (clone: 22C3) assessed on the basis of TPS was 100% **(**Fig. 1 **)**. The patient complained of back pain and left upper and lower limb paralysis which was found to be due to multiple bone metastases and hemorrhage from a massive brain metastasis **(**Fig 2a and b**)**. Her PS was 4. After undergoing palliative radiation therapy for the bone metastases and surgery followed by whole‐brain radiation therapy for the massive brain metastasis, her left upper and lower limb paralysis improved slightly, but her PS was still 3. Pembrolizumab (200 mg/bodyweight) was started as first‐line treatment, and a CT examination five months after the start of treatment revealed a partial response in all lesions, including the primary lesion, mediastinal lymph node metastases, liver metastasis, and brain metastasis **(**Fig. 2c‐f**)**. Two months after starting pembrolizumab, her PS had improved to 2.

## Discussion

Patients with advanced NSCLC and poor PS (PS ≥3) do not benefit from standard cytotoxic chemotherapy. However, *EGFR‐*mutation‐positive and ALK‐rearrangement‐positive NSCLC patients with poor PS often benefit from treatment with EGFR tyrosine kinase inhibitors (EGFR‐TKIs) and ALK tyrosine kinase inhibitors (ALK‐TKIs), which provide high activity with acceptable toxicity levels in patients with poor PS.^2^, ^3^ On the other hand, the efficacy of anti‐PD‐1 antibodies in frail patients has been limited, even when their tumors have been PD‐L1‐expression‐positive. Facchinetti *et al*. recently reported finding that the median progression‐free survival (mPFS) time and median overall survival (mOS) time of advanced‐NSCLC patients with a PS of 2 and high PD‐L1 expression level treated with pembrolizumab monotherapy was 2.4 months and 3.0 months, respectively, and poorer than the results reported by the KEYNOTE‐024 study, which evaluated the efficacy of pembrolizumab monotherapy in treatment‐naïve patients with advanced NSCLC and high PD‐L1 expression.^1^, ^4^ Additionally, the clinical outcome of pembrolizumab treatment was strongly dependent on PS assessed on the basis of disease burden and not on PS assessed on the basis of comorbidities.^4^ However, there have been no studies on the efficacy of PD‐1 blockade in patients with advanced NSCLC and poor PS (≥3), because it is extremely difficult to conduct clinical trials in these populations.

A post‐hoc analysis of the data obtained in the KEYNOTE‐010 study revealed the clinical outcomes of pembrolizumab treatment when PD‐L1 expression was further categorized into TPSs of 1%–24%, 25%–49%, 50%–74%, and ≥ 75%, and the results showed that increasing PD‐L1 expression was associated with more favorable outcomes of treatment with pembrolizumab.^5^ Aguilar *et al*. recently reported finding that advanced‐NSCLC patients with very high PD‐L1 expression (TPS 90%–100%) had a significantly higher objective response rate (ORR) and a significantly longer mPFS and mOS than those with a PD‐L1 expression TPS of 50%–89%.^6^ As stated above in regard to our own patient, the higher PD‐L1 pathway dependency of tumor immunity may be dispensable for a response to pembrolizumab in frail patients. Further analysis of the immune‐related tumor microenvironments in patients with advanced NSCLC, very high PD‐L1 expression (TPS 100%), and poor PS is needed.

Tumor mutation burden (TMB), defined as the total number of somatic mutations per coding area of a tumor genome, has been used as a predictive biomarker related to PD‐1 blockade across multiple solid tumors.^7^ On the other hand, the predictive value of TMB may not outweigh the value of PD‐L1 expression on tumor cells in advanced‐NSCLC patients. The combination of TMB plus PD‐L1 expression can serve as a useful predictive biomarker for the efficacy of PD‐1 blockade, because each is regarded as an independent predictor.^8^ However, assessments of TMB are based on biopsy or surgical specimens from solid tumors, and obtaining tissue biopsies could be challenging and invasive in advanced‐NSCLC patients, especially in frail patients.^9^ Cell‐free DNA (cfDNA) has been developed to assess TMB as a surrogate for biopsy specimens. However, high‐throughput molecular profiling of ctDNA remains technically challenging, and the optimal cutoff value for high TMB has yet to be determined. Additionally, clinical application of TMB is not currently feasible or expedient, because it is costly and time‐consuming, especially in frail NSCLC patients.^10^ Thus, PD‐L1 expression is still the only predictive biomarker in such patients.

In conclusion, we report a four‐case series of patients with advanced NSCLC and poor PS whose tumor had a high PD‐L1 expression level (TPS ≥50%), and only one case with a very high expression level (TPS 100%) responded to pembrolizumab. Although the number of cases in our series was very small, best supportive care is currently the only standard treatment available for advanced‐ NSCLC patients with poor PS (≥3) who do not have a driver mutation.^11^, ^12^ Moreover, no information is currently available regarding the efficacy of PD‐1 blockade in NSCLC patients with an extremely poor PS (≥3), because frail patients are excluded from clinical trials. We therefore concluded that pembrolizumab can serve as a treatment option for patients with poor PS, if theTPS for PD‐L1 expression on tumor cells is 100%. Additional studies in the future that include larger numbers of patients will be necessary to confirm our findings.

## Disclosure

Dr Yoshida received grants from Ono Pharmaceutical, Bristol‐Myers Squibb, AstraZeneca, and Takeda, and personal fees from AstraZeneca, Chugai, and Novartis. Dr Motoi received grants from NEC and Roche Diagnostics, and personal fees from MSD, Chugai, Novartis, and AstraZeneca. Dr Ohe reports having received grants and personal fees from Taiho, AstraZeneca, Chugai, Ono Pharmaceutical, Bristol‐Myers Squibb, Eli Lilly, MSD, Kyorin, Novartis, and Takeda, personal fees from Boehringer Ingelheim, Pfizer, grants from Kissei, Kyowa Hakko Kirin, Celtrion, Amgen, Nippon Kayaku, and grants from Dainippon‐Sumitomo, Ignyta, Janssen, and LOXO, outside the submitted work. The rest of the authors declare that they have no conflicts of interest.
